# A Case Report of Vaginal Delivery at Home due to Fear of Covid-19

**DOI:** 10.18502/ijps.v15i4.4306

**Published:** 2020-10

**Authors:** Mahnaz Nosratabadi, Nasrin Sarabi, Leila Masoudiyekta

**Affiliations:** 1 Department in Midwifery, School of Nursing and Midwifery, Dezful University of Medical Sciences, Dezful, Iran.; 2 Department of Nursing, School of Nursing and Midwifery, Dezful University of Medical Sciences, Dezful, Iran.

**Keywords:** *Covid-19*, *Fear*, *Home Delivery*

## Abstract

**Introduction:** Childbirth can be a normal and nonintervention process, but sometimes the process gets out of normal and requires immediate medical intervention. Thus, home delivery cannot be considered safe without coordination with the treatment staff. Sometimes fear of Covid-19 epidemic prevents mothers to go to the hospital for childbirth and they decide to do it in an unsafe condition, which puts the health of the mother and the neonate at risk.

**Presentation Case:** Our case was a pregnant woman with a negative blood group (A-) who did not come to the hospital because of fear of contracting Covid-19 from the hospital and decided to give birth at home without medical and midwifery support. After giving birth at home, she called the midwife who was taking care of her pregnancy. But she was still afraid to go to the medical center for postpartum care. The midwife informed the mother and her husband that they would be transferred to the midwifery clinic for further care and follow-up, with the necessary counseling and assurance of protective care to control Covid 19 transmission.

**Conclusion: **Counseling and training of protective methods during pregnancy can reduce the concerns of pregnant women. It is also recommended that pregnant women avoid unnecessary travel, public places, use of public transportation and contact with sick people, and most importantly, observe personal and public health issues. Some pregnant women may experience severe anxiety and depression during epidemics such as Covid 19, which require educational psychological counseling and continuous psychological support to prevent unintended consequences.

The novel coronavirus 2019 (COVID-19) is an epidemic in worldwide. Despite the progression of this epidemic, there is not enough information about the transmission of the disease to the fetus and baby yet (1).

The spread of fear as the infection increases have resulted in abnormal behaviors among individuals.

This is true of the Coronavirus, as it is a novel disease for which no definitive treatment has been found (2). In times like it, people feel fear, anxiety, and agitation due to constant media warnings about the outbreak.

In pregnancy, the immune system is partially suppressed (3). Pregnant women are susceptible to respiratory pathogens and severe pneumonia, which is why they are more susceptible to Coronavirus infection than other people. 

Therefore, pregnant women and newborn infants should be considered as high risk populations in strategies focused on the prevention and management of the infection (4). 

In the current state of epidemics of Covid-19, less attention has been paid to the psychological impact of the disease on pregnant women.

Thus, severe psychological distress may occur in pregnant women (5).

Some women decide to terminate their pregnancy due to fear of congenital infection and teratogenicity (3). During the epidemic of SARS disease, the use of central hospital services for pregnant women was reduced in some areas. Also, the number of Cesarean deliveries and the length of hospitalization were decreased (6). 

Some decide to give birth to childbirth at home. Although home delivery is associated with reduced cesarean section, and blood transfusions, some adverse maternal outcomes include prolonged delivery, the need to move to higher-level delivery management facilities, and more perineal rupture. On the other hand, some of the complications of home delivery that affect the baby include an increase in of low Apgar scores, neurological dysfunction and mortality (7).

The present study introduces a Rh negative pregnant mother who gave birth at home due to fear of Covid-19.

## Case Report

Our case was a 31-year-old pregnant woman, resident of Dezful, Khuzestan province, in her third pregnancy, 39 weeks and 4 days gestational age, with appropriate pregnancy control during pregnancy dated March 17, 2020, who due to severe fear of coronavirus 19 and refused to go the hospital. She eventually gave birth at home when labor pains began. She called her midwife after delivery in home and reported her history. According to the mother's history taken by the midwife, her postpartum hemorrhage and her general condition as well as the baby's condition were good. However, according to the midwife's knowledge of the negative blood group (A-) of the mother and the mother not satisfied with the transfer of the baby and herself to the hospital, the midwife asked the mother to transfer the fetal placenta to the midwifery clinic on recommended conditions to check the fetal cord blood sample. Her husband brought the placenta to the midwifery clinic, and cord blood sampling was performed. Fortunately, the placenta was also checked for completeness and the midwife ensured that the placenta was completely out. The fetal blood sample was A +and there was a need to inject RhoGAM for the mother. The mother was contacted and the mother and her husband were informed about the risk of not using RhoGAM, the necessity of a maternal examination, postpartum hemorrhage, and vital signs of the mother and infant, and infant vaccination. Self-care training for her and her infant against Coronavirus-19 transmission was given by phone, and her fear of illness was reduced after proper telephone counseling (Training and counseling were provided by midwives). To alleviate the mother's fear, the mother was told to go to the clinic when no other patient was in the midwifery clinic. The infant and mother were examined. RhoGAM injection was performed for the mother. Tetanus Immunoglobulin and other neonatal vaccinations were also administered. After follow-up, the mother was discharged from the clinic with normal complete blood count tests, normal postpartum hemorrhage, normal maternal and neonatal vital signs, and the ability to breastfeed.

## Discussion

With the increase in the number of people infected by the 2019 novel Coronavirus (COVID-19), (8) public anxieties and worries have been elevated in many regions, which is understandable. No one wants to get infected with a virus that has a relatively high risk of death (9). One psychological appearance of the COVID-19 pandemic is fear of childbirth among pregnant women (10). Fear of the disease (COVID-19) is growing due to false information and gossip. One of the reasons for fear of Covid- 19 is the bias and spread of inaccurate information about the disease worldwide. Thus, this misleading information replaces scientific evidence in people's minds and increases the risk of the disease. Judgment due to fear or misinformation causes fear among the public and creates a risk of trying to hold staff and health officials accountable. Above all, sometimes patients refuse to receive medical assistance (11). Fear of childbirth, fear of lack of control over the childbearing event, lack of knowledge, depression, and anxiety are believed to have significant roles in birth method selection. However, increasing the awareness of pregnant mothers can reduce their worries (12). According to research, the clinical symptoms of Covid-19 in pregnant women, such as cough, chest pain, shortness of breath, fever and lethargy, were not significantly different from non pregnant women. Also, scientists and researchers have not been able to confirm the vertical transmission of Coronavirus disease 2019 infection from the placenta within pregnancy, childbirth, and lactation. The results of research on normal vaginal delivery and cesarean section in pregnant women with coronavirus 2019 showed that birth methods did not affect the transmission of the disease to infants and all neonates were negative for Coronavirus disease 2019 infection (13). Complications of coronavirus 2019 in pregnant women are less than H1N1 influenza in 2009. Pregnant women who were exposed to the H1N1 influenza in 2009 were at higher risk for preterm labor, emergency Cesarean delivery, ICU hospitalization, and death (14). In February 2020, many reports were published in The Lancet that indicated mental health care should be available in the health care system, and more awareness is needed to better control stress in cases of unexpected diseases. Prenatal and postpartum mental health of pregnant women should be a priority in health care system programs because of their impact on the physical and mental health of pregnant women, fetuses, infants, and their families (15).

We, as a universal society, need association to stop the prevalence of Covid-19. Bright and clear information on the prevalence of the disease globally and principled warnings by experts in each region are essential to decrease concern. Psychologically, when conditions and living environments change, people feel insecure, discomfort, and worried. When the reason of the pandemic is unclear, misconceptions and unreal often appear. Even humans are blamed for no reason (16). Measures and surveys widely performed in medical centers and laboratories are another reason for the epidemic of fear and anxiety among people (17).

Many people think that viruses are hovering in the air for a long time and they are afraid of getting sick from these viruses, and this belief makes them very worried and anxious, and sometimes they even try to label the groups as the culprits of this disease. We try to eliminate this fear and concern (16).

Therefore, there is a need to design an effective antistigma and antifear program to break the misperception about Covid-19, increase public's knowledge about Covid-19, and encourage positive and supportive messages (18). 

## Limitation

Our access to studies similar to home births and fears of Covid-19 was very limited. Also, no studies on Covid-19 fear reduction intervention were found in relation to maternal mental health. Therefore, it is recommended that intervention studies be performed to reduce the fear and anxiety of pregnant mothers about Covid-19.

## Conclusion

Often, pregnant women are physically and mentally at risk. One of the important actions of the health care system in conditions of diseases that threaten the physical and mental health of people, especially pregnant women, is planning to raise awareness and reduce the stress and fear of pregnant mothers. Counseling and protective training during pregnancy can eliminate the concerns of pregnant mothers. Some pregnant women may experience severe anxiety and depression, which requires psychological support to avoid unpleasant consequences. Also, in epidemic conditions of infectious diseases, maternal education by the health team can reduce the fear of childbirth in the hospital and prevent unwanted negative consequences for mothers and infants in certain situations.
